# The Small World of Adult Hippocampal Neurogenesis

**DOI:** 10.3389/fnins.2018.00641

**Published:** 2018-09-20

**Authors:** Rupert W. Overall, Gerd Kempermann

**Affiliations:** ^1^German Center for Neurodegenerative Diseases (DZNE), Dresden, Germany; ^2^Center for Regenerative Therapies Dresden, Technische Universität Dresden, Dresden, Germany

**Keywords:** transcriptomics, genetics, gene networks, genetic reference panel, stem cells

## Abstract

Making mechanistic sense of genetically complex biological systems such as adult hippocampal neurogenesis poses conceptual and many practical challenges. Transcriptomics studies have helped to move beyond single-gene approaches and have greatly enhanced the accessibility to effects of greater numbers of genes. Typically, however, the number of experimental conditions compared is small and the conclusions remain correspondingly limited. In contrast, studying complex traits in genetic reference populations, in which genetic influences are varied systematically, provides insight into the architecture of relationships between phenotypes and putative molecular mechanisms. We describe that the correlation network among transcripts that builds around the adult neurogenesis phenotype and its endophenotypes is, as expected, a small-world network and scale free. The high degree of connectivity implies that adult neurogenesis is essentially an “omnigenic” process. From any gene of interest, a link to adult hippocampal neurogenesis can be constructed in just a few steps. We show that, at a minimum correlation of 0.6, the hippocampal transcriptome network associated with adult neurogenesis exhibits only two “degrees of separation.” This fact has many interesting consequences for our attempts to unravel the (molecular) causality structure underlying adult neurogenesis and other complex biological systems. Our article is not written with the expert on network theory in mind but rather aims to raise interest among neurobiologists, active in neurogenesis and related fields, in network theory and analysis as a set of tools that hold great promise for coping with the study of “omnigenic” phenotypes and systems.

## Introduction

The mammalian hippocampus is a brain structure with a key role in learning and memory. It is also unusual in harboring a population of neural stem cells that can be activated by environmental cues to generate new additional granule cell neurons, both at a baseline level and on additional functional demand (Kempermann et al., 2015). Adult hippocampal neurogenesis persists throughout life and can be conceptualized as consisting of several distinct developmental sub-processes such as stem cell recruitment and proliferation, migration of lineage-determined progenitor cells, maturation and neurite outgrowth, synaptogenesis and functional integration, and so on (Kempermann et al., 2004, 2015; Toni et al., 2008; Overall et al., 2012; Bonaguidi et al., 2016; Dulken et al., 2017). Each of these component processes is under distinct genetic control resulting in a diverse constellation of genes regulating adult neurogenesis as a whole (Kempermann et al., 2006; Overall et al., 2012).

The genetic complexity of this system is extreme and this makes determining the genes and molecular pathways underlying the normal function of adult neurogenesis extraordinarily difficult. To overcome this limitation we have, over the last decade, applied approaches from the growing field of systems genetics to our subject in order to investigate the interactions between genes and phenotypes. The central idea is to exploit the immense statistical power in genetic reference populations. A genetic reference population is a collection of lines or strains with defined, and well-characterized, genetic background which can be used to identify the contributions of genes to measured traits. It should be noted, that the term “trait,” as used in this manuscript, refers generally to any *measurable* feature of the biological system. This is in contrast to the traditional sense of *observable* feature. Thus, for the mouse model described, possible traits can include physiological, behavioral, and histological variables. In fact, even gene expression levels can be considered a “trait,” as these measurements can be analyzed in exactly the same way as traditional traits; and this is the approach taken in the present study. Using one such resource in mice, in 2006 one of us (GK) published a study describing four cellular traits (based on histology) relating to adult hippocampal neurogenesis (Kempermann et al., 2006). As might be expected for a system under complex genetic control, these traits did not have strong associations to a single locus in the genome, suggesting there is no one single gene that governs their expression. But when the traits were related to whole brain gene expression data, a number of co-varying candidate transcripts could be discerned. Since then, new resources and tools have become available, which now allow us to re-analyze these data to provide deeper insight into the genetic control of adult hippocampal neurogenesis. The current study presents the results of such analyses and uses this example to discuss the technological advances as well as the open questions.

## Results

### Genetic Reference Populations as Model of Genetic Diversity

There have been astonishing advances made by reductionist genetics, and several projects are making impressive progress in systematically perturbing all known genes in the mouse (for example^1^). Nevertheless, such attempts at understanding genetic control of complex traits are limited as they do not take into account the combinatorial effects of multiple genes. Polygenic effects are often not additive so that the dizzyingly vast number of possibilities makes such an undertaking wholly intractable. In addition, any combination containing lethal mutations cannot be studied.

There are, however, other approaches that are based on investigating combinations of naturally occurring genetic variability. Genetically diverse populations contain large numbers of essentially randomly segregating alleles providing a sort of “shotgun” combination of genetic variants. The task then becomes to dissect the effects of any particular gene or subset of genes. This can be done if the number of genotypes is sufficiently high and the phenotyping error low. For this approach there are good tools available including genome-wide association studies [GWAS; which also recently celebrated a 10th birthday (Visscher et al., 2017), a technique that has been successfully used to map risk genes for common sporadic diseases, such as Parkinson’s disease (Nalls et al., 2014)], and quantitative trait locus (QTL) mapping. Although the mathematical details differ, both GWAS and QTL mapping aim to match the quantitative trait data to the genotype at different positions across the genome. If a certain allele (a genomic variant) is consistently associated with higher or lower trait measurements, then this suggests that a gene at or near that position in the genome could be causing the difference in trait expression. These are powerful techniques to investigate potential links between genes and measurable phenotypic or clinical outcomes.

Over the last decade we have focused our attention on a particularly powerful genetic population in mice; the BXD recombinant inbred panel (Taylor, 1978; Taylor et al., 1999; Peirce et al., 2004). This genetic reference population is derived from two very well characterized laboratory mouse strains, C57BL/6J (on which the mouse reference genome is based) and DBA/2J (the oldest existing fully inbred strain). Both of these parental strains have been fully sequenced. The BXD panel has been derived by crossing the F1 hybrids of these two strains and inbreeding the resulting F2 progeny. In fact, additional recombinations have been fixed in many of the strains by also including several rounds of intercrossing prior to inbreeding (Peirce et al., 2004). The resulting panel thus contains very many recombination events fixed by sibling mating over more than 20 generations to yield inbred lines – hence the designation as a “recombinant inbred” population. Advantages of such a model include genetic tractability (there are only two alleles segregating, albeit in highly shuffled combinations) and reproducibility (the inbred nature of the lines means that an unlimited supply of animals of each genotype can be phenotyped). An additional implication is that, because the lines remain stable over time, new traits can be matched to existing ones allowing reanalysis and extension of historical datasets – as we have done for the current report.

Because the strains have been genotyped, association of a trait with the genotype at any locus can be calculated, allowing potentially causal genes to be identified. In addition, correlation between different traits measured across the population can suggest links between the traits themselves which may be due to commonalities in their genetic regulation.

### Adult Hippocampal Neurogenesis Is a Polygenic Process

**FIGURE 1:**
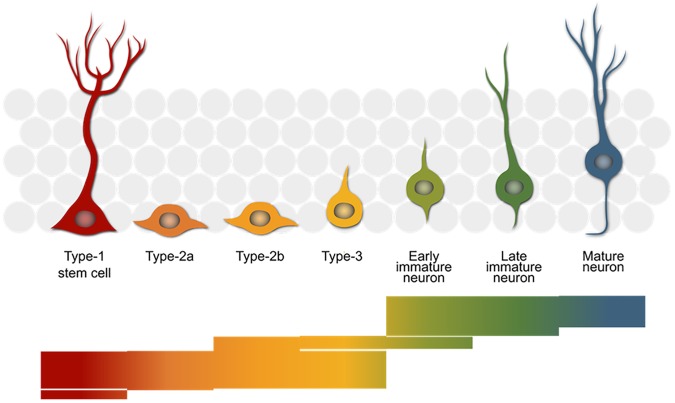
The neurogenic trajectory in the adult hippocampus. The stem cells (red) give rise to rapidly proliferating progenitor cells (orange–yellow) that finally exit the cell cycle (green) and mature into new-born neurons (blue), which integrate into the surrounding granule cell network (gray). The bars below reflect the number of genes with a reported role in the neurogenic process. Each of the 390 stacked lines making up the image represents one entry in the MANGO database (http://mango.adult-neurogenesis.org) covering the stages in which a single gene is known to have a certain function.

Our biological process of interest is adult hippocampal neurogenesis – the generation of new neurons from a stem cell population in the adult mammalian hippocampus. This development is comprised of several sub-processes (**Figure 1**) which appear to be under distinct genetic control (Kempermann et al., 2006) and which in total require the concerted action of many genes (Overall et al., 2012). The stem cells divide to produce rapidly proliferating precursor cells (type 2) and these become less proliferative and more neuron-like (type 3) as they differentiate along the neurogenic trajectory. Eventually, after around a week, the new-born cells exit the cell cycle and become immature neurons and finally, if they survive, mature to become fully functional granule cells integrated into the surrounding neural network.

Performing histology with an endogenous marker of proliferating cells, such as Ki67, a bulk quantification of all the cycling cells (types 1, 2, and 3) can be obtained. In addition, by labeling cells in S-phase with a thymidine analog, it is possible to mark all cells which were proliferating at the time of labeling. When histology is performed 4 weeks later, after all labeled cells have left the cell cycle, it is possible to measure the number of surviving cells (typically around 50% of all new-born precursor cells die before reaching the mature neuron stage). Further co-staining the surviving new-born cells with endogenous markers of mature neurons or astrocytes, the rate of production of these cell types can be calculated. This methodology was applied previously to measure adult hippocampal neurogenesis in 31 strains of the BXD panel (Kempermann et al., 2006). The phenotypes measured were Ki67-positive proliferating cell number (PROL), thymidine analog-retaining surviving new-born cells (SURV), new-born neurons (NEUR), and new-born astrocytes (ASTR). The data are available from the web resource GeneNetwork^2^ under the BXD phenotype IDs 10795–10798. All of the traits exhibit a wide range of expression across the different strains (summarized in **Table 1**) and a strong genetic basis for this variance is evidenced by the generally high estimates of heritability; 0.53 (PROL), 0.68 (SURV), 0.70 (NEUR), 0.23 (ASTR) (Kempermann et al., 2006), which implies that (except in the case of ASTR) the majority of the trait variance can be explained by the genotype of the mice rather than individual or environmental influences. Despite the clear role of genetic background, the roles of individual genes is less clear. To date, over 250 genes have been implicated in some aspect of adult hippocampal neurogenesis (Overall et al., 2012). Are all of these genes essential for control of the system? Do they all act on distinct features or do they all act together? It is more likely that genetic control is a complex mix of these extremes.

**Table 1:** A summary of the published neurogenesis traits.

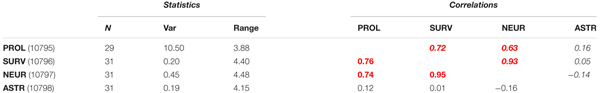

*Shown are summary data for the four neurogenesis traits described in the text including the number of samples (*N*), the variance (var), and the range (given as standard deviations between the highest and lowest strains). Also shown are the correlations between traits (Pearson’s *r* in the lower triangle and Spearman’s *ρ* in italics in the upper triangle). Very strong correlations are highlighted in red.*

We can discover genes with potential links to our trait of interest by calculating the correlation of the trait against expression of all the genes in a tissue sample. This was done in the original study using whole brain transcript abundance estimates, but can now be performed using a more relevant dataset we have since generated in which dissected hippocampal mRNA from 69 BXD strains was hybridized to Affymetrix M430v2 microarrays (Overall et al., 2009). Transcripts with the strongest correlation with a trait of interest are expected to be more directly involved in genetic control of the trait. **Table 2** shows the top 20 transcripts correlating with each of the neurogenesis traits. We can see from **Figure 1** and **Table 1** that many of the same genes correlate with each of the different traits, indicating that they share some common genetic control. But to get a better picture of which regulatory pathways are shared between traits and which are specific, we need to look at the data in a different way.

**Table 2:** Top 20 correlating transcripts for each of the neurogenesis traits.

	PROL		SURV		NEUR		ASTR	
				
	Symbol	*r*	Symbol	*r*	Symbol	*r*	Symbol	*r*
1	Zfp771	0.72	Gcat	0.82	Vsig8	0.77	Lig1	0.71
2	Arvcf	0.68	Fitm1	0.79	Gcat	0.76	Nlgn3	0.64
3	Krt7	0.68	Krt7	0.77	Gm9905	0.76	Tet2	0.62
4	Dgat2	0.67	Dxo	0.76	Krt7	0.76	Rbm33	0.61
5	Mzf1	0.66	Vsig8	0.76	Fitm1	0.76	Arhgap35	0.59
6	Tnks1bp1	0.66	Il17rc	0.76	Pmf1	0.74	Meaf6	0.56
7	Atxn2l	0.66	Oas1f	0.74	Dxo	0.73	Rad54b	0.56
8	Capn10	0.65	2310050C09Rik	0.74	2700049A03Rik	0.72	Igkv1-117	0.56
9	Tuba3a	0.64	Gm9905	0.72	Mroh4	0.71	Srpk1	0.56
10	Arhgdia	0.64	Pmf1	0.72	Actl7b	0.71	Spaca1	0.56
11	Cad	0.64	Ece2	0.71	Amh	0.7	Srrm3	0.55
12	Gm9905	0.64	Mroh4	0.7	Oas1f	0.7	Mapk8ip1	0.55
13	Kcns1	0.63	2700049A03Rik	0.7	Nrtn	0.7	Rreb1	0.55
14	Panx3	0.63	Tsfm	0.7	Tsfm	0.7	AA386476	0.54
15	Aamp	0.63	AY761184	0.69	AY761184	0.7	Cdkal1	0.54
16	Ifrd2	0.63	Amh	0.69	Cort	0.7	Col6a1	0.54
17	Rcc1	0.63	Nrtn	0.69	Txnrd2	0.7	Sae1	0.54
18	Tfip11	0.63	Acrbp	0.69	Fam229a	0.7	Tmigd1	0.53
19	Myo18a	0.63	Cort	0.68	Nfkbib	0.69	0610030E20Rik	0.53
20	Ccdc12	0.63	Iqcd	0.68	Rax	0.69	Hap1	0.53


*Each of the neurogenesis traits was correlated to all hippocampal transcripts and the 20 strongest correlations are shown.*

### Networks as Tools for Complexity

**FIGURE 2:**
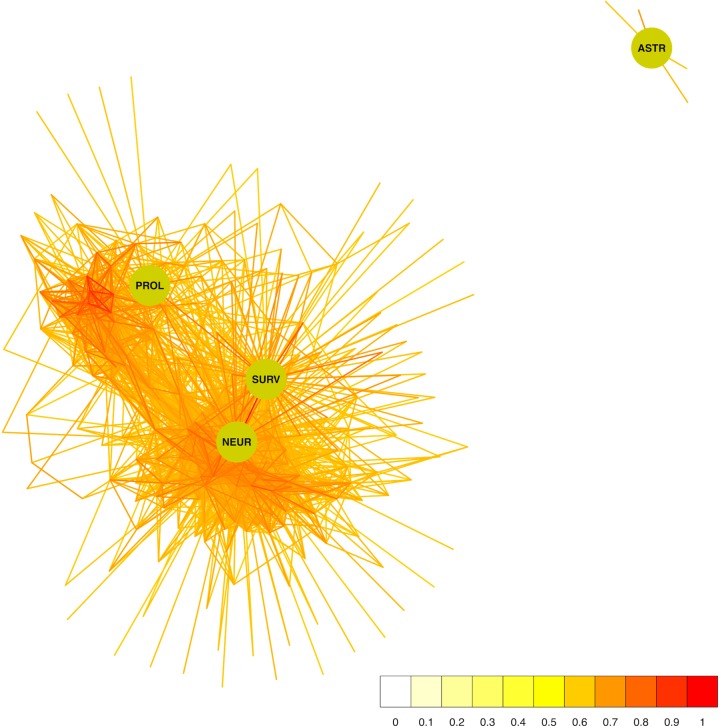
The network of genes surrounding the adult neurogenesis traits. The four traits are depicted as green circles and the connections to correlating transcripts (*r* ≥ 0.6) are drawn in a color representing the correlation strength. The transcript nodes are left undrawn.

One appealing approach to study the interactions of many genes simultaneously is to view them as a network. Formally, a network (or “graph” as it is known in mathematics) is a collection of entities (genes or histology traits in our case) which can be connected in some way (for example by sharing similar expression profiles). **Figure 2** shows a network constructed from the neurogenesis-related traits and all genes correlating with any one of them with a Pearson’s *r* of at least 0.6. Pearson’s correlation coefficient (*r*) is a measure of how similar the expression patterns of two variables (traits/genes) are. Although two traits may be correlated through indirect causality (e.g., both are under the control of a third trait) and there is a certain “background” influence of all other traits on the traits being correlated, the fact that two traits share a similar pattern of expression reveals the presence of some link and is a useful tool in the dissection of functional genetic interactions. Visualized in this way, it is immediately obvious that ASTR is under quite distinct genetic control while the other traits share many correlating transcripts, with SURV and NEUR (not surprisingly) being most similar.

Using this network, we can also ask which genes might be more likely to be part of a regulatory pathway by identifying transcripts which not only correlate to the histology traits but also to each other (at the same threshold of *r* ≥ 0.6). These nodes in the network will have high measures of “centrality,” meaning that they are connected to more of the other nodes. The more neighbors (or “friends”) a node has, the more likely it is to be influential in the network, so nodes with a high centrality score (often called “hubs”) could be important modulators of tissue function. Various centrality scores are presented in **Table 3** which suggest several potential key genes in the regulation of adult hippocampal neurogenesis.

**Table 3:** Gene centrality in the adult neurogenesis network.

	Degree		Betweenness		Eigenvector		Closeness		PageRank	
					
	Symbol	Score	Symbol	Score	Symbol	Score	Symbol	Score	Symbol	Score
1	Rpl18	75	Pmf1	605	Rpl18	0.83	Pmf1	0.149	Rpl18	0.013
2	Pmf1	73	Rpl18	473	Snai3	0.82	Ccdc13	0.148	Snai3	0.012
3	Snai3	72	Tmem134	422	Pmf1	0.82	Snai3	0.148	Pmf1	0.012
4	Prap1	65	Sppl2b	348	Prap1	0.77	Prap1	0.148	Prap1	0.011
5	Kif12	62	Pold4	337	Slc41a3	0.75	Slc41a3	0.147	Kif12	0.011
6	Tmem134	61	Snai3	327	Gsdmcl2	0.74	Rpl18	0.147	Gsdmcl2	0.011
7	Slc41a3	61	Ccdc13	319	Tmc8	0.73	Fitm1	0.147	Slc41a3	0.011
8	Tmc8	61	Prap1	313	Krt78	0.72	Tmem134	0.147	Tmem134	0.011
9	Gsdmcl2	61	Leng9	302	Ccdc13	0.72	Gsdmcl2	0.147	Tmc8	0.010
10	Krt78	60	Pacsin3	296	Cblc	0.71	Pold4	0.147	Krt78	0.010


*Several measures of node centrality were calculated for the network surrounding the adult neurogenesis traits at a correlation of *r* ≥ 0.6.*

### Correlation Networks Are “Omnigenic”

When interpreting expression correlation networks, it is important to be aware that the correlation is a mathematical property, and not a biological one. The adage “correlation does not imply causality” is very true here, and there are several reasons why two phenotypes might exhibit co-varying expression. For example, two genes might be correlated because they are both under the direct control of a third gene. In a whole-tissue sample such as used here, it is also possible that two genes are strongly correlated because they are expressed in a certain cell type and the abundance of that cell type could be regulated by a gene in a different cell. Nevertheless, correlations are likely to be indicative of some shared or indirect causality. Unlike other types of approaches, network analyses enable such shared underlying causalities to be potentially identified.

**FIGURE 3:**
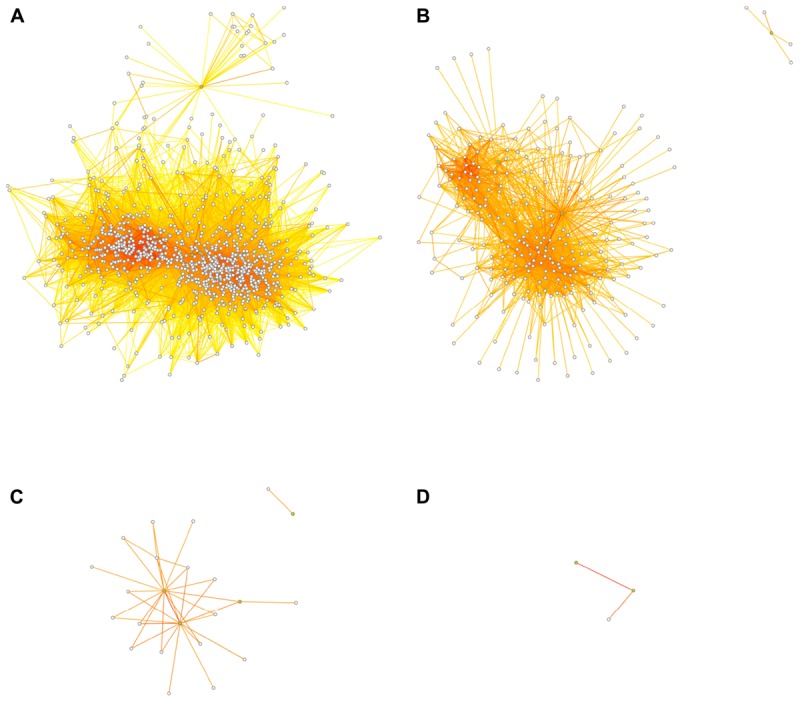
Dissociation of the network at higher thresholds. Threshold choice alters network structure, as fewer connections survive the increasingly stringent filter. At thresholds of *r* ≥ 0.5 **(A)**, *r* ≥ 0.6 **(B)**, *r* ≥ 0.7 **(C)**, or *r* ≥ 0.8 **(D)**, the transcript network surrounding the adult neurogenesis traits falls apart so that at the highest threshold no transcript nodes are present and the “network” becomes uninformative. The four neurogenesis trait nodes are depicted in green, the transcript nodes are white. Transcript nodes with no connections to other nodes are not shown. Edge coloring is as in **Figure 2**.

While a true direct causal relationship (A causes B; where A might be a transcription factor binding the promoter of B, for example) ought to be reflected in a strong correlation, an indirect relationship (such as B correlating with C; where both B and C are similarly regulated by A but not directly interacting with each other) can also lead to above-threshold correlations. Of course, many weaker correlations may also arise purely by chance. As the threshold for inclusion into the network is lowered, more such indirect or spurious connections will be present. As the threshold tends to zero (or -1 in a network based on signed Pearson correlations), the connectivity of the network tends toward completeness, i.e., everything connected to everything. At the extremes, a threshold of zero means that all nodes are connected to all other nodes and the number of connections equals (*n*^2^-*n*)/2, where *n* is the number of nodes; at a maximum threshold of 1, on the other hand, nodes can only be perfectly correlated to themselves, so there are no connections between nodes at all. **Figure 3** shows the effect of different thresholds on the 1st-level (direct connections) network surrounding the neurogenesis-related traits.

This behavior is different from most other biological network types, where connections are based on a biological property and might vary only within the ranges of measurement error. Protein–protein binding networks are a good example, in which a connection represents the binding between protein species. Nevertheless, even in the absence of measurement error, molecular interactions may extend far beyond typically accepted pathway boundaries (Boyle et al., 2017).

**FIGURE 4:**
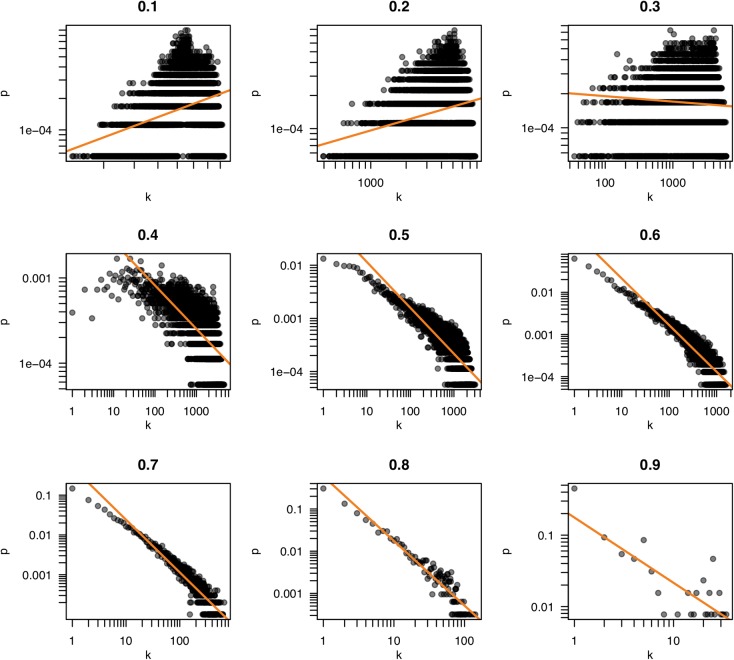
Scale freedom of the hippocampal transcriptome network. The number of neighbors of each node (k) and the probability of nodes having k neighbors (p) are plotted for the correlation thresholds 0.1–0.9. The log-log plots of these values reveal a linear relationship when a network exhibits “scale free” behavior. Scale freedom is a property seen in most biological networks. Because the true biological network underlying a correlation network is expected to exhibit this behavior, a threshold where a linear relationship is first seen could indicate an appropriate threshold selection.

Because the properties of a network are dependent on the pattern of connections, the selection of a threshold becomes a consequential task. Often, the criterion of “scale freedom” is used to decide if a network conforms to expected norms. The “scale-free” property of biological networks simply means that the structure of the network is very uneven. In a very simple network, such as a lattice or a random network, every node is connected to roughly the same number of other nodes (neighbors). Such a network is homogeneous and the number of connections per node tends to scale in proportion to the network size. In scale-free networks, on the other hand, the number of neighbors is very different for different nodes; some have very many friends, some have very few, following what is termed a “power law” distribution. This can be observed by counting the number of neighbors (the “degree”) of each node and seeing how likely it is that any particular node has a certain number of neighbors (the “degree probability”). When plotted together, these values in scale-free networks exhibit a log-log relationship (very few very rich and very many very poor). **Figure 4** shows plots of the degree distribution for the whole hippocampal expression network filtered at various thresholds. The relationship between degree and degree probability clearly breaks down at very high and low thresholds but appears stable between values of 0.6 and 0.8. Thus our choice of *r* ≥ 0.6 for the network in **Figure 2** is a good compromise that maximizes the size of the network assayed while remaining in this scale-free range.

### The Small World of Adult Neurogenesis

Even when a restrictive threshold is applied, the scale-free nature of the network means that the minimal “distance” between any two nodes is smaller than would be expected if the network were randomly connected. This is referred to as the “small world” property of networks – a term that was inspired by a famous experiment by Milgram (1967) and Travers and Milgram (1969) that asked how many intervening acquaintances would be required to pass a letter by hand across the United States. The number turned out to be six. This finding inspired a play by John Guare, whose title “Six Degrees of Separation” became emblematic for the paradigm. In the hyper-connected computer age, Facebook has since brought this number down to 3.57^3^. A related concept is the Erdős number, relating any scientific author to prolific mathematician Paul Erdős based on whether two authors have published together (GK, for example, has an Erdős number of 4, RWO of 5). It is worthwhile noting here that the use of the word “degree” in this popular sense is not the same as the graph-theoretical meaning introduced above. These “degrees of separation” are really related to the length of the shortest path connecting two nodes (in fact they equal the shortest path length minus one).

Watts and Strogatz (1998) proposed the “small world” model, characterized by the clustering of nodes and short separations of clusters via individual nodes in those clusters (“clustered connectedness”). In effect, this model says that the clustering around highly connected nodes (known as *hubs*) means that every node is not very far from a hub, and thus it is more likely that two nodes are connected via a hub. In other words, the average length of the *shortest path* is reduced. They also made the surprising discovery that this network structure is very commonly found in real-world networks; such as social and biological networks. This influential work turned out to be the starting point for studies that increasingly revealed how the underlying network structures determine the properties of a system (Vespignani, 2018). Network theory evolved from just a descriptive tool to an explanatory approach in complex contexts.

**FIGURE 5:**
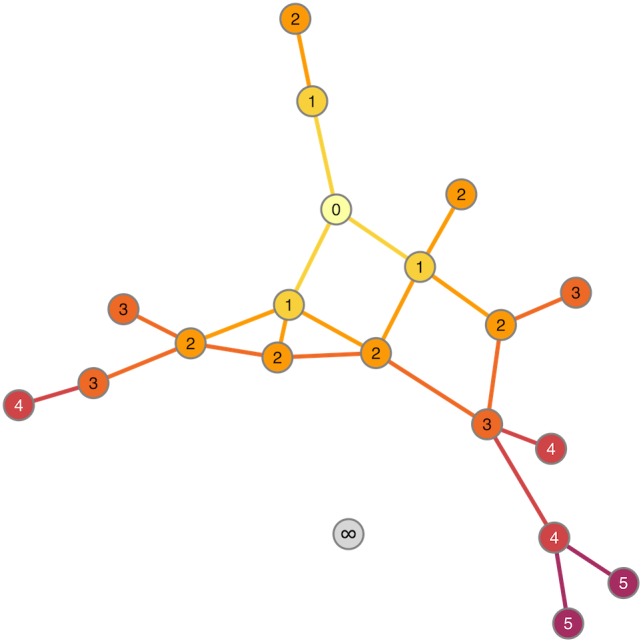
Degrees of separation. Starting from the “seed” node (numbered 0), the nodes and edges are colored according to how many steps it takes to reach them. Thus, nodes labeled “1” are 1 step (0 intermediaries = 0 “degrees of separation”) from “0,” nodes labeled “2” are 2 steps (1 intermediary) and so on. The gray node (labeled “∞”) is not connected to the node “0” at all. It is unreachable from that node so cannot be included in the degrees of separation calculation.

**FIGURE 6:**
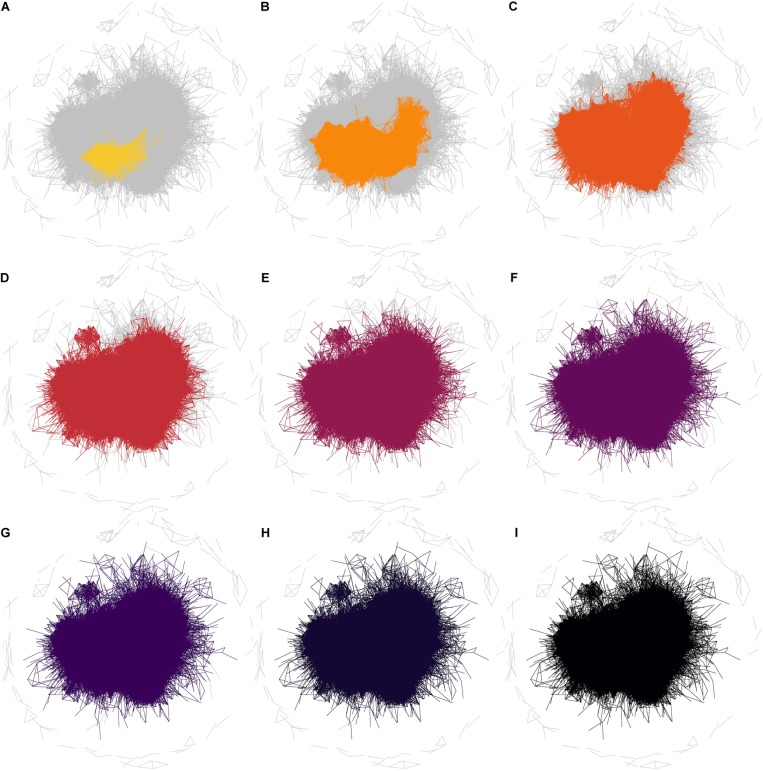
Degrees of separation in the whole correlation network. Networks are shown for the phenotype NEUR at a correlation threshold of *r* ≥ 0.6. In these plots, all edges for the full correlation network (at *r* ≥ 0.6) are drawn in gray and then the edges connecting other nodes to NEUR by one step are colored **(A)**. The colored subnetwork is then extended to also include nodes connected by two steps (one “degree of separation”) to NEUR **(B)**. Subsequent steps are shown in the other panels **(C–I)**. It is clear that the entire (reachable) network is essentially already covered by 3 steps; i.e., almost all nodes are within two degrees of separation from the NEUR phenotype. The edges not connected by any path (unreachable edges) remain gray in this plot.

The observation that the neurogenesis-associated transcription network is small-world means that the average number of steps required to join any two traits (i.e., the number of intermediate nodes in the shortest path) is again smaller than would be expected in a randomly connected network. The implication is that the “friends-of-friends” network extending out from the adult neurogenesis-related traits will spread to cover a rapidly increasing number of nodes. The example network in **Figure 5** demonstrates the concept of “degrees of separation” by showing how distant nodes in a network can be connected by intermediate steps. Thus gene 1 might influence expression of gene 2 which might influence gene 3, and so on. In this way, despite a direct correlation between gene 1 and gene 3 not being detectable, its effect can spread, or “percolate,” throughout the network via intermediaries. In **Figure 6**, the percolation effect of the NEUR trait can be seen by highlighting the 1st-level neighbors in the first panel, and the 2nd-, 3rd-level, etc., in consecutive panels until the entire transcriptome has been covered (or at least the whole connected subnetwork – some nodes are not reachable and so can never be connected to the neurogenesis traits regardless how many steps are used, see **Figure 5**). But otherwise it is clear already from **Figure 6** that it does not require many steps to cover the entire transcriptome network. Visualization of this effect using such traditional network plots is poor, however, as the edges rather than the nodes are drawn and there is a lot of overdrawing in a densely connected network. This results in the much discussed “hairball” problem in the visualization of complex networks.

**FIGURE 7:**
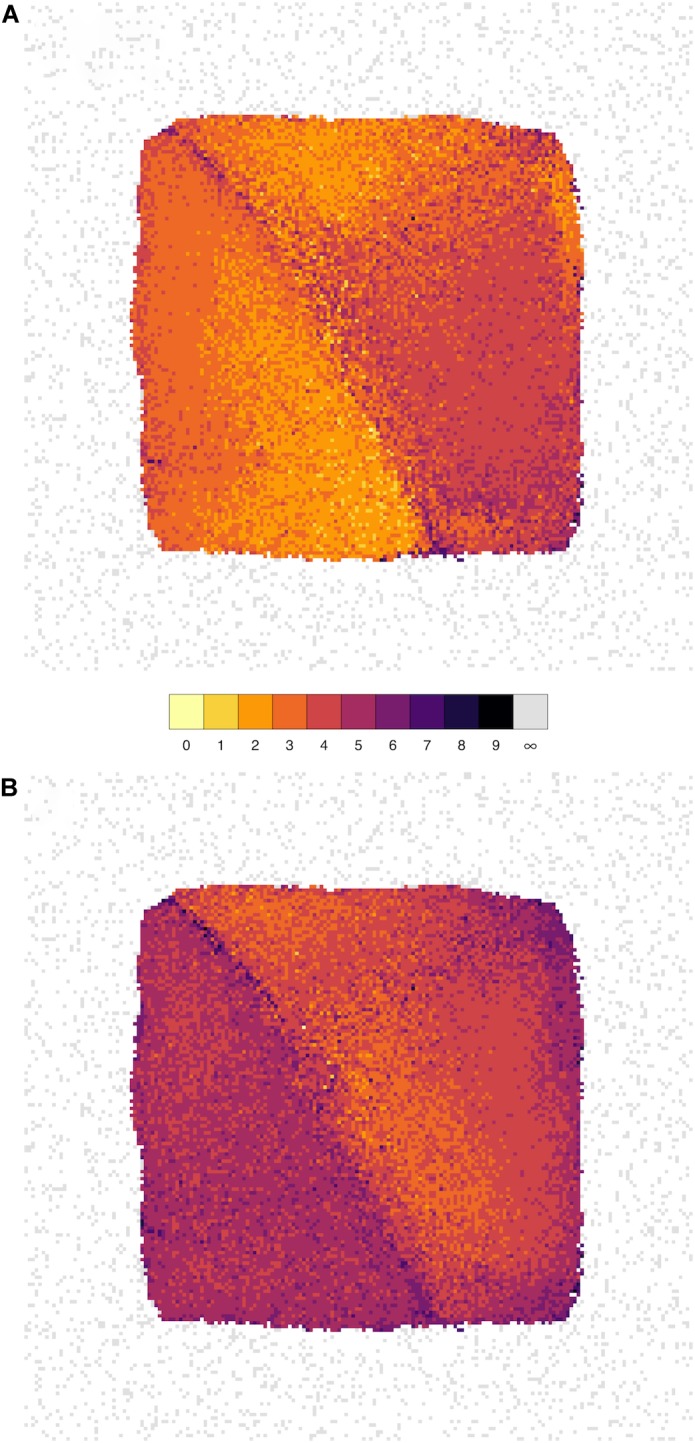
The influence of genes spreads rapidly throughout the network. Network grid plots are shown for the phenotypes NEUR **(A)** and ASTR **(B)** at a correlation threshold of *r* ≥ 0.6. In these plots, all nodes of the full correlation network (at *r* ≥ 0.6) are laid out on a grid such that the distance between them reflects the correlation (more strongly correlated nodes/traits are closer together). The node layout is identical in both plots. Nodes are colored by how closely they are connected to the seed nodes (NEUR or ASTR). The first-level neighbors (i.e., nodes directly connected to the seed node) are shown in bright yellow (1 in the color key), second-level neighbors (“neighbors-of-neighbors” with a shortest path length of 2) in yellow, and so on. Unconnected nodes are shown in gray and tend to get pushed to the periphery of the plot by the layout algorithm. It can be clearly seen by the brighter colors that NEUR is mode highly connected in this network than ASTR and its influence spreads to cover the entire network in fewer steps.

We have therefore devised a novel layout which arranges all of the nodes on a grid such that the distance between the cells on the grid reflects the correlation; nodes with stronger correlation tend to be closer together. Starting from the NEUR trait, there are 163 1st-level correlating traits (see **Figure 2** and **Table 4**), shown in yellow in **Figure 7**. The 3222 2nd-level correlates are in pale orange and so on until the entire reachable network is covered within nine steps. A summary for all four neurogenesis traits is shown in **Table 4**.

**Table 4:** Numbers of neighbors at different degrees of separation.

	Number of neighbors			
**Level**	**PROL**	**SURV**	**NEUR**	**ASTR**
1	56	138	163	4
2	1890	2897	3222	63
3	7568	8770	9187	1809
4	13498	13783	14067	7790
5	15078	15146	15175	14062
6	15333	15335	15339	15253
7	15376	15376	15376	15367
8	15381	15381	15381	15380
9	15382	15382	15382	15382


*For each of the neurogenesis traits (in the four columns), the number of transcript nodes connected at a correlation threshold of *r* ≥ 0.6 are shown in the first row (“Level 1”). The subsequent rows show the number of transcripts connected to the transcripts from the previous level. The numbers in the “NEUR” column correspond to the number of nodes connected by highlighted links in the panels of **Figure 6**.*

It is also possible to calculate the minimum number of links (edges) required for any gene to reach any other gene (the length of the *shortest path* between the nodes). At a threshold of *r* ≥ 0.6, the average of the shortest paths between all pairs of genes is only 3.16, implying that any gene might be exerting an effect on any other gene with only around two intermediates. Thus, the gene network surrounding adult hippocampal neurogenesis at this threshold approximates two degrees of separation.

## Conclusion

Our research is continuing to identify influential genes affecting the various aspects of adult hippocampal neurogenesis and asking how the many implicated genes work together to shape the fate of the stem cells and their progeny. But the presented results highlight that the combination of phenotyping and whole transcriptome expression profiling in genetic reference populations, together with now readily-available network analysis tools, can yield a very different view of biology than used to be possible. It becomes clear from a glance at the networks presented above that a reductionist program of genetics will hit difficulties at some point. In particular, studies based on only two genotypes (knockout vs. wild-type, for example) assess effects of the mutation on the phenotype that is the result of a complex alteration of the underlying genetic network. This is the reason why knockout experiments might have different results in different genetic backgrounds.

Consequently, from adding together the information of single-gene studies, only very limited insight into the detailed genetic network architecture underlying the trait can be gained. Our BXD study on adult neurogenesis revealed a heritability of the new neuron phenotype of 0.7 but, despite this high value, the QTL analysis did not reveal any significant loci. Instead, a large number of weaker associations were observed suggesting a role for many genomic loci with only small influence on the trait. These minor associations, in sum, reflect the underlying genetic network and imply the presence of many genes with individually small effects but which together shape expression of the trait. This is an example of the “missing heritability” that has been reported for many other traits (Manolio et al., 2009). Human body height is a much publicized example where, even taking an enormous number of polymorphisms into account, still less than half of the variance in the population can be explained (Yang et al., 2010). Network-based analyses and visualization at the very least expose this complexity and provide tools to dissect the problem in ways not accessible by the traditional approaches that still prevail in molecular neuroscience today. The use of networks to aid analysis is not restricted to the methods presented above. For example, structural equation modeling [SEM; see (Kline, 2015) for a recent overview], whose predecessors were actually invented in a genetic context, has become a key technique in other disciplines such as psychology and sociology but deserves broad re-introduction to biology. The key feature of SEM is that it can be used to test causality models involving latent variables – that is variables that cannot be directly measured or are conceptual. Adult neurogenesis as a process has many such latent properties and might even be considered a latent variable itself. Combining QTL mapping with SEM can further help in dissecting out the genetic regulation of highly complex traits (Li et al., 2006).

The highly connected nature of molecular biological pathways means that a systems-level *Weltanschauung*, the consideration of genes in the context of their molecular environment, together with the corresponding bioinformatics toolkit will be more often called for over the next decade of biology and beyond. The “two degrees of separation” of the molecular network is not only a mathematical analytical concept but reflects the connectivity (and hence structure) of the underlying biology. Perturbation of a gene, by experimental manipulation or in disease, is likely to have much wider effects than might at first be predicted. This idea has important consequences for drug development, gene therapy safety and the handling of genetic diseases. But it also fundamentally changes what we consider as a “genetic” or “molecular mechanism” underlying complex traits and phenotypes. In fact, our work suggests that the influence of single genes might extend far wider than generally thought.

The example presented here demonstrates that teasing useful information out of complex networks can be started today by any researcher in biomedicine, and that we, as a field, might have to rethink our range of methodologies to achieve complex goals. This progress should not, and ultimately cannot, be deferred to the experts of systems biology – systems thinking must, and will, go mainstream. As the technologies to deal with complex data mature and the appreciation of genes as parts of networks becomes more widely established, it will surely be seen that all traits are “omnigenic” to some degree. But with tools to manage the complexity, this information will only help us to better understand the role of genes in shaping biologically important traits.

## Materials and Methods

### Trait Summary Statistics and Heritability

The adult hippocampal neurogenesis traits are from a previous study (Kempermann et al., 2006) and are available from the GeneNetwork BXD phenotypes database under the accessions 10795–10798. The summary statistics in **Table 1** were directly retrieved from the GeneNetwork records for the traits. The heritability scores, h^2^, of the traits are likewise taken from this previous study.

### Preparation of Transcript Expression Data

The transcript expression data used for the examples in this manuscript are derived from a previously published Affymetrix M430v2 microarray dataset (Overall et al., 2009) that has been remapped to ENSEMBL gene identifiers using a custom CDF from the Brainarray project^4^. For this work, the version 22 CDF^5^ was further processed using custom code to remove probes containing a polymorphism between C57BL/6J and DBA/2J. The R package used for this, CDFSnipeR, is available from http://research.rupertoverall.net/resources/cdfsniper/. The microarray data were processed, using this custom CDF, with the R package *affy* using the RMA normalization algorithm as implemented by the function *justRMA*.

### Correlation Network

Transcript and phenotype data were used to generate a correlation matrix using Pearson’s *r*. Networks were derived from this by thresholding the *r* values (*r* ≥ 0.6 if not otherwise stated). The R package *igraph* was used for the analyses presented in **Table 3**. The plots for scale freedom in **Figure 4** were prepared in R using base functions.

### Network Graph Layout Algorithms

Layout of the graphs for visualization was performed by either the R package *igraph* (**Figures 2**, **3**, **5**) or, for the large graphs in **Figure 6**, by a custom algorithm implemented in a new R package, DataNet. This algorithm uses a simplified force-directed approach to iteratively optimize edge distances, and scales well in performance for dense networks of this size. The package is available from http://research.rupertoverall.net/resources/datanet/. The graphs in **Figure 7** were created using a novel algorithm, GridGraph, to optimally arrange nodes on a constrained grid. The code is available as an R package from http://research.rupertoverall.net/resources/gridgraph/.

## Data Availability

The datasets analyzed for this study can be found in GeneNetwork (http://www.genenetwork.org).

## Author Contributions

RO and GK designed the study, provided the data and tools, and wrote the manuscript. RO performed the analyses.

## Conflict of Interest Statement

The authors declare that the research was conducted in the absence of any commercial or financial relationships that could be construed as a potential conflict of interest.

## Footnotes

1 http://www.mousephenotype.org/

2 http://genenetwork.org

3 https://research.fb.com/three-and-a-half-degrees-of-separation/

4 http://brainarray.mbni.med.umich.edu/Brainarray/Database/CustomCDF/genomic_curated_CDF.asp

5 http://mbni.org/customcdf/22.0.0/ensg.download/Mouse4302_Mm_ENSG_22.0.0.zip
